# Nasopharyngeal Carriage of Antimicrobial-Resistant Pneumococci in an Intensively Sampled South African Birth Cohort

**DOI:** 10.3389/fmicb.2019.00610

**Published:** 2019-03-27

**Authors:** Rendani I. Manenzhe, Clinton Moodley, Shima M. Abdulgader, F. J. Lourens Robberts, Heather J. Zar, Mark P. Nicol, Felix S. Dube

**Affiliations:** ^1^Division of Medical Microbiology, Faculty of Health Science, University of Cape Town, Cape Town, South Africa; ^2^National Health Laboratory Service, Groote Schuur Hospital, Cape Town, South Africa; ^3^Department of Paediatrics and Child Health, Red Cross War Memorial Children’s Hospital and SA-MRC Unit on Child and Adolescent Health, University of Cape Town, Cape Town, South Africa; ^4^Department of Molecular and Cell Biology, Faculty of Science, University of Cape Town, Cape Town, South Africa

**Keywords:** pneumococcus, nasopharyngeal carriage, infants, antibiotic-resistance, carriage duration

## Abstract

**Introduction:** Nasopharyngeal (NP) colonization by *Streptococcus pneumoniae* (pneumococcus) precedes the development of respiratory tract infection. Colonization by antimicrobial-resistant pneumococci, especially in infants, is a major public health concern. We longitudinally investigated antimicrobial-resistance amongst pneumococci colonizing the nasopharynx of South African infants immunized with the 13-valent pneumococcal conjugate vaccine (PCV13).

**Methods:** NP swabs were collected every second week from birth through the first year of life from 137 infants. Pneumococci were identified and serotyped using conventional microbiological techniques, and their antibiotic susceptibility profiles determined by disk diffusion and *E*-test.

**Results:** All infants were immunized with 3 doses of PCV13. 1520 pneumococci (760 non-repeat) isolates were recovered from 137 infants; including non-typeable (*n* = 99), PCV13 (*n* = 133) and non-PCV13 serotypes (*n* = 528). The prevalence of penicillin, erythromycin, and cotrimoxazole non-susceptibility was 19% (95% CI 17–22%) (3% fully resistant), 18% (95% CI 15–21%) (14% fully resistant), and 45% (95% CI 42–49%) (36% fully resistant), respectively. The predominant penicillin-non-susceptible serotypes included 19A, 19F, 15B/15C, 15A, and 21, while susceptible serotypes included 23A, 34, and 17A. Multidrug-resistance (MDR) was observed in 9% (95% CI 7–11%) of the isolates. PCV13 serotypes were more likely to be non-susceptible, compared to non-PCV13 serotypes, to penicillin (26% vs. 16%, *p* = 0.007), erythromycin (23% vs. 15%, *p* = 0.027) and cotrimoxazole (62% vs. 41%, *p* < 0.001). Non-susceptibility to penicillin, erythromycin, and cotrimoxazole remained relatively constant through the first year of life (*X*^2^ test for trend: *p* = 0.184, *p* = 0.171, and *p* = 0.572, respectively). Overall, penicillin or erythromycin-non-susceptible pneumococci were carried for a shorter duration than susceptible pneumococci [penicillin (mean days, 18 vs. 21, *p* = 0.013) and erythromycin (mean days, 18 vs. 21, *p* = 0.035)]. Within individual infants carrying the same serotype longitudinally, changes in antibiotic susceptibility were observed over time in 45% (61/137) of infants and these changes were predominantly for penicillin (76%, 79/104).

**Conclusion:** Prevalence of NP carriage with antibiotic-non-susceptible pneumococci was relatively constant throughout the first year of life. PCV13 serotypes were more commonly non-susceptible to penicillin, erythromycin, and cotrimoxazole. Penicillin or erythromycin-non-susceptible pneumococci were carried for a shorter duration than penicillin or erythromycin-susceptible pneumococci.

## Introduction

Nasopharyngeal (NP) colonization by antimicrobial-resistant *Streptococcus pneumoniae* (pneumococcus) is a global public health concern (Ginsburg and Klugman, 2017). The pneumococcus is an important bacterial cause of childhood pneumonia (O’Brien et al., 2009; Andrade et al., 2018). NP colonization with pneumococci is a prerequisite for development of pneumococcal disease (Bogaert et al., 2004). The nasopharynx of children serves as a natural reservoir for pneumococci and the source for person to person transmission (Schrag et al., 2000). Vaccination with pneumococcal conjugate vaccine (PCV) is effective in reducing both vaccine type pneumococcal carriage and invasive disease (Flasche et al., 2011). Despite this, carriage prevalence has remained unchanged due to an increase in carriage of non-vaccine serotypes (Simell et al., 2012).

A rise in antibiotic-resistance among pneumococci has reduced the effectiveness of empiric antibiotics used to treat pneumococcal infections (Zhao et al., 2012). Beta-lactam antibiotics are commonly used for the treatment of infections caused by pneumococci (Liñ Ares et al., 2010). Widespread beta-lactam prescription and routine immunization exert selective pressures on the pneumococcal population structure which contribute to the emergence of beta-lactam resistant-pneumococci (Schrag et al., 2000; Liñ Ares et al., 2010). This has in turn resulted in the increased use of other classes of antibiotics such as macrolides and fluoroquinolones. Transmission of pneumococci that are resistant to these classes of antibiotics is also increasing (Lin et al., 2016).

Detection of antibiotic-resistance is important for successful therapy in the individual child, as well as for tracking antibiotic-resistance patterns, which inform empiric treatment guidelines, and antimicrobial stewardship (Kim L. et al., 2016). Most studies on the NP carriage of antibiotic-resistant pneumococci are cross sectional, and there are few longitudinal studies describing pneumococcal resistance patterns, including duration of carriage of susceptible and resistant pneumococci especially in low and middle income country settings where there is the greatest burden of disease (Ekdahl et al., 1997; Meropol et al., 2016). This study aimed to investigate antimicrobial-resistance patterns in pneumococci colonizing the nasopharynx of PCV-13 vaccinated South African infants, from birth through the first year of life.

## Materials and Methods

### Study Population and Sampling

NP swabs were collected fortnightly from 137 infants enrolled between May 29th 2012 and May 31st 2014 as part of intensive cohort of the Drakenstein Child Health Study (DCHS), a longitudinal, prospective birth-cohort study in the Drakenstein sub-district, Cape Town, South Africa (Zar et al., 2014). The study population is a stable, semi-urban community with a low socioeconomic status. This area also has a high incidence of childhood pneumonia (incidence 0.29 episodes/child year) (Zar et al., 2016). Pregnant women (>18 years) were enrolled in public sector health clinics during the second trimester, and followed until childbirth. Thereafter, infants were enrolled at birth and followed through their first year of life (Zar et al., 2014). All births occurred at Paarl hospital, a single public hospital serving this area. All 137 infants received a 2+1-dosing schedule of PCV-13 at 6 weeks, 14 weeks, and 9 months of age, according to the South African Expanded Program on Immunization (EPI-SA, 2015). For this study we included the first 137 infants enrolled in the cohort who had the most complete fortnightly NP sampling (defined as at least 23–26 fortnightly collected NP swabs).

### Bacterial Isolates

The NP sample collection, transportation, culture and storage have been described previously (Dube et al., 2018). Pneumococcal isolates were serotyped using sequetyping and confirmed by Quellung (Dube et al., 2015) and stored in 1 ml skim milk-tryptone-glucose-glycerol (STGG) medium at -80°C for further batch processing. Isolates were resuscitated by inoculating 20 μl of thawed STGG onto 2% sheep blood agar (Green Point Media Laboratory, National Health Laboratory Service, Cape Town, South Africa) and incubated for 24–48 h at 37°C, in 5% CO_2_.

### Antibiotic Susceptibility Testing (AST)

Susceptibility testing of the isolates to oxacillin (1 μg), erythromycin (5 μg) and cotrimoxazole (1.25–23.75 μg) (bioMérieux, Marcy I’Etoile, South Africa) was performed using the disk diffusion method and interpreted according to 2017 guidelines (Clinical and Laboratory Standards Institute [CLSI], 2017). Oxacillin non-susceptible isolates were further characterized using benzylpenicillin/penicillin G minimum inhibitory concentrations (MIC), determined by *E*-test (bioMérieux, Marcy I’Etoile, South Africa) according to the manufacturer’s instructions, and interpreted using 2017 guidelines (Clinical and Laboratory Standards Institute [CLSI], 2017). A subset of 243 randomly selected isolates were screened for resistance to ciprofloxacin (5 μg) and levofloxacin (5 μg) (bioMérieux, Marcy I’Etoile, South Africa). *S. pneumoniae* ATCC 49619 and *Staphylococcus aureus* ATCC 25923 were used as quality control strains. Oral penicillin breakpoints were used to interpret penicillin results (susceptible: ≤0.06 μg/ml, intermediate: 0.12–1 μg/ml, resistant: ≥2 μg/ml). Intermediate (low-level resistance) and resistant (high-level resistance) isolates were all considered as non-susceptible isolates. Multidrug-resistance was defined as non-susceptibility to the three classes of antibiotics (penicillin, erythromycin, and cotrimoxazole) tested and dual-resistance as non-susceptibility to two classes of antibiotics. To calculate the prevalence of non-susceptible isolates, longitudinal isolates from a single infant with the same antibiogram and serotype were classified as a single isolate. The acquisition of a non-susceptible pneumococcal strain was defined as the detection of a non-susceptible strain for the first time in an infant or the detection of a non-susceptible strain that was initially susceptible. Loss of a non-susceptible strain was similarly defined. The acquisition of a non-susceptible pneumococcal strain was presumed to start at the mid-point between the last sampling time-point before which the non-susceptible strain was detected and the time-point at which the non-susceptible strain was first identified, whilst a loss of a non-susceptible strain was considered as the mid-point between the time at which the non-susceptible strain was last identified and the next time-point. The carriage duration was determined by the difference between the loss date and acquisition date (Dube et al., 2018).

### Ethics Statement

This study was carried out in accordance with the recommendations of the Human Research Ethics Committee of the Faculty of Health Sciences, University of Cape Town. The protocol was approved by the Human Research Ethics Committee of the Faculty of Health Sciences, University of Cape Town (HREC refs: 401/2009 and 740/2013) and the Western Cape Provincial Child Health Research Committee. All subjects gave written informed consent in accordance with the Declaration of Helsinki.

### Statistical Analysis

Statistical analyses were performed using STATA software (Stata Corporation, College Station, TX, United States). Chi-square and Fisher’s exact tests were used where applicable to compare the differences in the prevalence of carriage of non-susceptible pneumococci. Unpaired *t*-test (mean-comparison test) was used to compare the mean carriage durations. A two-tailed *p*-value of <0.05 was considered statistically significant.

## Results

### Participant Demographics

Of 137 infants included in this study, 56% (77/137) were female and 6% (8/137) were preterm. The majority of infants were born via vaginal delivery (80%, 109/137), followed by emergency cesarean section (13%, 18/137), and elective cesarean section (7%, 10/137). Only 7% (10/137) of the infants were admitted to a ward after birth whereas 93% (127/137) roomed with their mother. Overall, 25% (34/137) of infants were born to HIV-infected mothers, with only one infant being HIV-infected (Table 1). PCV13 immunization coverage was 100% at 6 weeks, 14 weeks, and 9 months scheduled visit, respectively. Despite this, immunization was delayed by at least 2 weeks in 6% (8/137) and 18% (24/137) of the infants at 6 weeks and 9 months, respectively.

**Table 1:** Characteristics of infants included in this study.

Characteristics	% (Total), *N* = 137
**Gender:**	
Female	56 (77)
**Mode of delivery:**	
Vaginal	80 (109)
Emergency cesarean	13 (18)
Elective cesarean	7 (10)
Preterm (<37 weeks)	6 (8)
Low birth weight (<2500 g)	13 (18)
HIV exposure^#^	24 (33)
**Admission after birth:**	
Ward	7 (10)
Roomed with the mother	93 (127)
Breastfed before discharge	89 (119)
**PCV13 immunization**	
6 weeks^∗^	100 (137)
14 weeks	100 (137)
9 months^∗∗^	100 (137)


*^#^One of the 33 HIV-exposed infants was HIV-infected. ^∗^Immunization delayed by at least 2 weeks in 8 infants, ^∗∗^Immunization delayed by at least 2 weeks in 24 infants.*

### Antibiotic Susceptibility Patterns Among Non-repeat Isolates

Fifty percent (760/1520) of isolates were non-repeat pneumococcal isolates. Of these, 49% [376/760, (95% confidence interval, CI 46–53%)] were susceptible to all three antibiotics tested (penicillin, erythromycin, and cotrimoxazole). In total, 19% [147/760, (95% CI 17–22%)], 18% [136/760, (95% CI 15–21%)], and 45% [344/760, (95% CI 42–49%)] were non-susceptible to penicillin, erythromycin and cotrimoxazole, respectively (Supplementary Table S1). However, 3% [21/760, (95% CI 2–4%)], 14% [108/760, (95% CI 12–17%)], and 36% [274/760, (95% CI 33–40%)] of the non-susceptible isolates were fully resistant to penicillin, erythromycin, and cotrimoxazole, respectively. None of the 243 randomly selected isolates were non-susceptible to ciprofloxacin or levofloxacin.

### Serotype Distribution and Antibiotic Susceptibility Patterns

**FIGURE 1:**
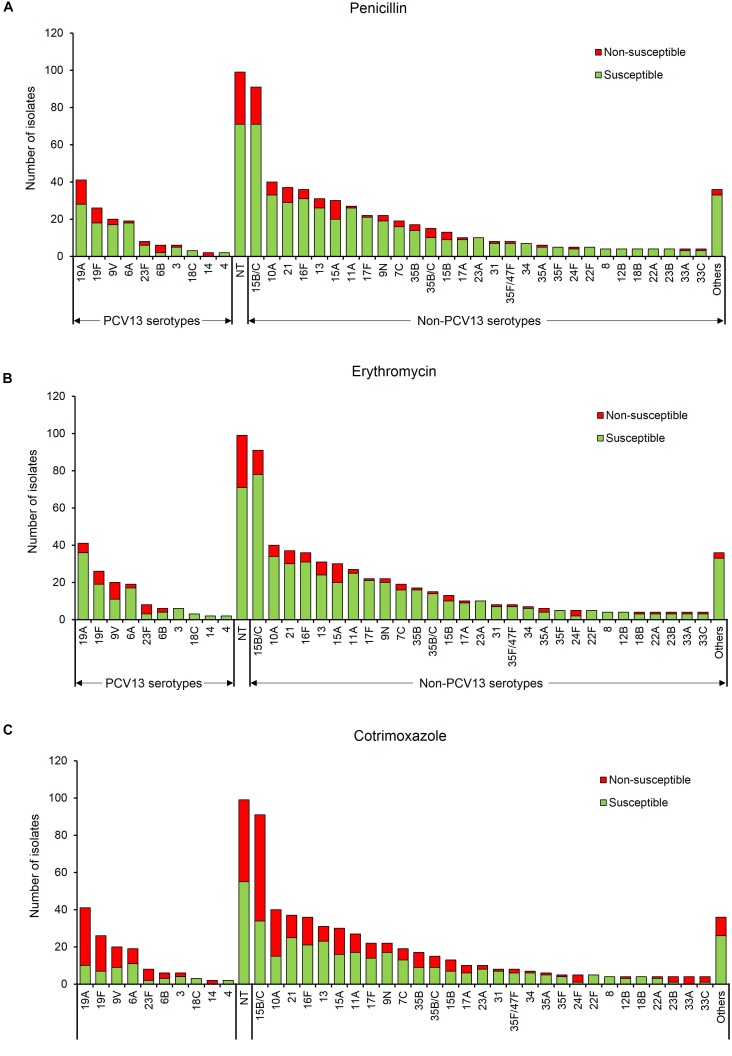
Serotype frequency and susceptibility profiles of 760 non-duplicate pneumococcal isolates, by serotype. Susceptibility to **(A)** penicillin, **(B)** erythromycin, and **(C)** cotrimoxazole. Others include serotypes 20, 38, 11F, 18A, 19B, 19C, 22A/F, 23B, 25A/38, 33B/35C, 33F, 47F, and 6C/D. PCV13 serotypes- serotypes included in the 13-pneumococcal conjugate vaccine, Non-PCV13 serotypes- serotypes not included in the 13-pneumococcal conjugate vaccine, NT, Non-typeable.

Non-PCV13 serotypes (*n* = 528) were more commonly isolated than PCV13 serotypes (*n* = 133). However, PCV13 serotypes were more likely to be non-susceptible than non-PCV13 serotypes to penicillin, erythromycin and cotrimoxazole (Figure 1, Table 2, and Supplementary Table S1). The rates of dual resistance and multidrug-resistance (MDR) were low (Table 2 and Supplementary Table S1). A significantly higher proportion of PCV13 serotypes had dual penicillin-cotrimoxazole resistance and dual erythromycin-cotrimoxazole resistance, compared to non-PCV13 serotypes. The overall prevalence of MDR was 9% [68/760, (95% CI 7–11%)], which was higher (but not significant) for PCV13 than non-PCV13 serotypes (Table 2 and Supplementary Table S1). The most frequent MDR PCV13 serotypes were 19F (6%, 4/68) and 19A (4%, 3/68), while non-PCV13 serotypes included 15B/15C (15%, 10/68), 16F (6%, 4/68), 13 (6%, 4/68), and 15A (6%, 4/68) (Supplementary Table S1). The predominant penicillin-non-susceptible pneumococci were non-typeable isolates (19%, 28/147), serotypes 15B/15C (14%, 20/147), 19A (9%, 13/147), 15A (7%, 10/147), 19F (5%, 8/147), and 21 (5%, 8/147). Of note, penicillin-non-susceptible isolates were frequently also non-susceptible to erythromycin (61%, 89/147) or cotrimoxazole (80%, 118/147).

**Table 2:** Proportion of antibiotic-non-susceptible pneumococcal isolates by vaccine type.

Antibiotic(s)	% Non-susceptible isolates (95% confidence interval)		*P-*value^a^
		
	PCV13	Non-PCV13	
	serotypes	serotypes	
	(*n* = 133)	(*n* = 528)	
Penicillin	26 (19-34)	16 (13-19)	0.007
Erythromycin	23 (16-30)	15 (12-18)	0.027
Cotrimoxazole	62 (53-70)	41 (37-46)	<0.001
Penicillin + erythromycin	2 (0-5)	3 (2-4)	0.532
Penicillin + cotrimoxazole	12 (8-19)	5 (4-8)	0.003
Erythromycin + cotrimoxazole	11 (6-17)	4 (2-6)	0.001
MDR	10 (6-16)	7 (5-10)	0.244


*^*a*^Difference between PCV13 vs. Non-PCV13 serotypes. PCV13 serotypes, Serotypes included in the 13-pneumococcal conjugate vaccine; Non-PCV13 serotypes, Serotypes not included in the 13-pneumococcal conjugate vaccine. MDR, Multidrug-resistance.*

### Antibiotic-Non-susceptibility Trends Over-Time

**FIGURE 2:**
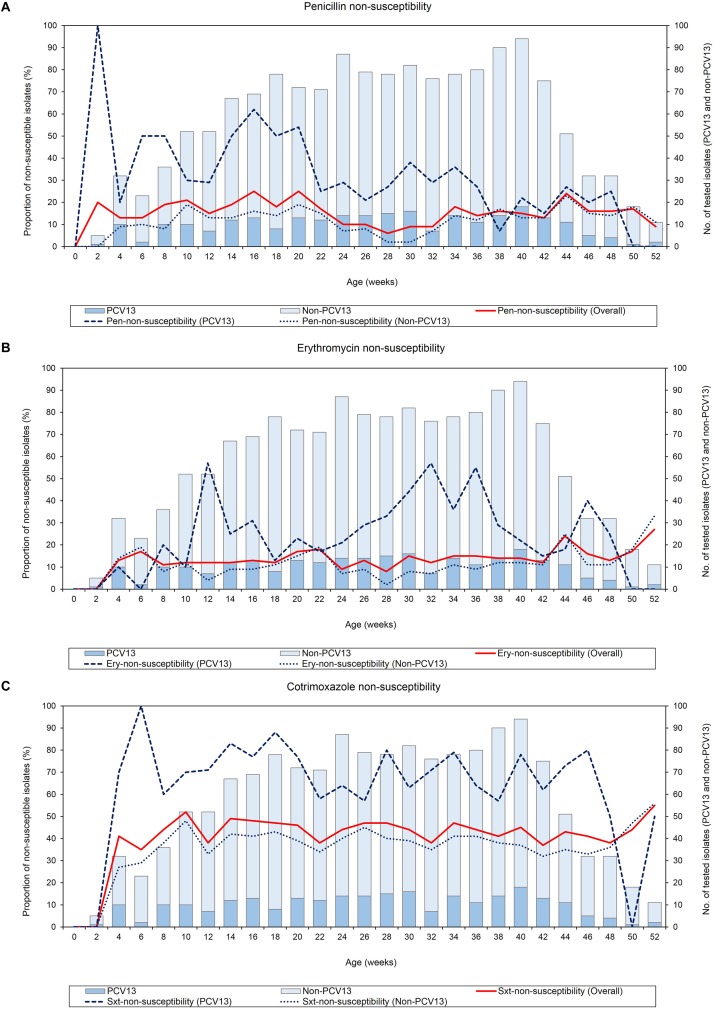
Point-prevalence of antibiotic-non-susceptible pneumococci (*n* = 1520) obtained from 137 infants throughout the first year of life, **(A)** penicillin-non-susceptibility, **(B)** erythromycin-non-susceptibility, and **(C)** cotrimoxazole-non-susceptibility. Pen, Penicillin; Ery, Erythromycin; Sxt, Cotrimoxazole; No, Number; PCV13 serotypes, Serotypes included in the 13-pneumococcal conjugate vaccine; Non-PCV13 serotypes, Serotypes not included in the 13-pneumococcal conjugate vaccine.

None of the infants were colonized by pneumococci at birth, thereafter, antibiotic-non-susceptible pneumococci were detected from week 2 throughout the first year of life (Figure 2). NP carriage of penicillin-non-susceptible pneumococci was observed as early as 2 weeks of age, however, erythromycin- and cotrimoxazole-non-susceptibility were not observed until week 4, where non-susceptibility was 13% (95% CI 4–29%) and 41% (95% CI 24–59%), respectively. Although there was a slight decline in penicillin-non-susceptibility between weeks 22 to 32, the levels of non-susceptibility to penicillin, erythromycin, and cotrimoxazole remained relatively constant through the first year of life (*X*^2^ test for trend: *p* = 0.184, *p* = 0.171, and *p* = 0.572, respectively) (Figure 2). Non-susceptibility to more than one antibiotic among the pneumococcal serotypes is shown in Supplementary Figure S1. Although prevalence of non-susceptibility to more than one antibiotic was generally low, the frequency of dual resistance to penicillin-cotrimoxazole was higher during the first 6 months and then declined over the remaining 6 month (*X*^2^ test for trend, *p* < 0.001). There were no other clear trends for changes in the prevalence of dual or multidrug-resistance over time.

### Relationship Between Antibiotic Non-susceptibility and Duration of Carriage

The mean carriage duration for all pneumococcal serotypes ranged from 13 to 65 days; the mean carriage duration for individual serotypes has been previously described (Dube et al., 2018). Overall, penicillin or erythromycin-non-susceptible pneumococci were carried for a shorter duration than susceptible pneumococci, and the difference in the mean carriage duration was significant: penicillin-non-susceptible (18 days, 95% CI 17–20) vs. penicillin-susceptible (21 days, 95% CI 20–22), *p* = 0.013 and erythromycin-non-susceptible (18 days, 95% CI 16–21) vs. erythromycin-susceptible (21 days, 95% CI 20–22), *p* = 0.035. Cotrimoxazole-non-susceptible and -susceptible pneumococci were carried for similar durations (21 days, 95% CI 19–22, and 20 days, 95% CI 19–22, respectively, *p* = 0.766) (Table 3).

**Table 3:** Carriage duration of antibiotic-non-susceptible pneumococcal isolates by vaccine type.

Antibiotic susceptibility	Carriage duration, mean number of days (95% confidence interval)				*P*-value^a^
		
	Any serotype	Non-typeable	PCV13 serotypes	Non-PCV13 serotypes	
**Penicillin**					
Non-susceptible	18 (17–20)	16 (13–23)	20 (18–23)	18 (15–19)	0.313
Susceptible	21 (20–22)	17 (15–20)	19 (17–21)	22 (21–23)	0.033
**Erythromycin**					
Non-susceptible	18 (16–21)	15 (14–16)	23 (17–29)	17 (14–20)	0.037
Susceptible	21 (20–22)	17 (15–18)	20 (18–22)	22 (21–24)	0.086
**Cotrimoxazole**					
Non-susceptible	21 (19–22)	16 (14–17)	22 (19–25)	21 (19–23)	0.649
Susceptible	20 (19–22)	17 (15–18)	17 (14–20)	21 (20–23)	0.030


*^*a*^ Difference between PCV13 vs. Non-PCV13 serotypes. PCV13 serotypes, Serotypes included in the 13-pneumococcal conjugate vaccine; Non-PCV13 serotypes, Serotypes not included in the 13-pneumococcal conjugate vaccine.*

When stratified according to serotype, penicillin or erythromycin-non-susceptible non-PCV13 serotypes were carried for a shorter duration than susceptible non-PCV13 serotypes: penicillin (mean days, 18 vs. 22, respectively, *p* = 0.019) and erythromycin (mean days, 17 vs. 22, respectively, *p* = 0.007) (Table 3). These findings were reversed for PCV13 serotypes; non-susceptible PCV13 serotypes were carried for a longer duration than susceptible PCV13 serotypes but this was not statistically significant for penicillin or erythromycin: penicillin (mean days, 20 vs. 19, respectively, *p* = 0.621), erythromycin (mean days, 23 vs. 20, respectively, *p* = 0.122), and cotrimoxazole (mean days, 22 vs. 17, respectively, *p* = 0.027). These difference were largely due to serotypes 19F and 9V (PCV13 serotypes) which were carried for a longer duration if non-susceptible: serotype 19F, for penicillin and both serotypes 19F and 9V, for erythromycin (Supplementary Figure S2).

### Changes in Antibiotic Susceptibility Pattern Within the Same Infant

**FIGURE 3:**
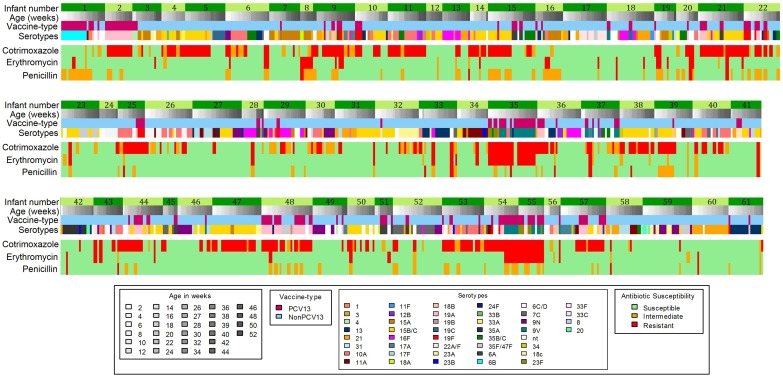
Longitudinal carriage of pneumococci showing shifts in antibiotic-non-susceptibility profiles which occurred in 61 out of 137 infants. PCV13 serotypes, Serotypes included in the 13-pneumococcal conjugate vaccine; Non-PCV13 serotypes- Serotypes not included in the 13-pneumococcal conjugate vaccine.

Changes in antibiotic susceptibility profiles within the same pneumococcal serotype carried by an infant were observed in 45% (61/137) of infants (Figure 3). Of the 104 shifts in susceptibility profiles observed in these infants, 76% (79/104) were for penicillin, including changes from susceptible to non-susceptible (*n* = 41) and non-susceptible to susceptible phenotypes (*n* = 38) (Figure 3).

## Discussion

This study reports the antimicrobial-non-susceptibility patterns of pneumococci colonizing the nasopharynx of PCV13 vaccinated South African infants. Despite high vaccine coverage, PCV13 serotypes were identified in 54% of children and these were more commonly non-susceptible to penicillin, erythromycin, and cotrimoxazole than non-PCV13 serotypes. The point-prevalence of antibiotic-non-susceptible pneumococci to penicillin, erythromycin, and cotrimoxazole was relatively constant from week 4 through the first year of life. Overall, penicillin or erythromycin-non-susceptible pneumococci were carried for a shorter duration than penicillin or erythromycin-susceptible pneumococci.

There are limited data on the NP carriage of antibiotic-non-susceptible pneumococci among vaccinated children during the first year of life (Coles et al., 2002; Hill et al., 2006; Russell et al., 2006). The prevalence of penicillin-non-susceptible pneumococci was 19% in this study. The rate of non-susceptibility was higher compared to that reported in India (3.4%) (Coles et al., 2002), Fiji (11.4%) (Russell et al., 2006) and The Gambia (13.3%) (Hill et al., 2006) among infants. The majority of the isolates in the present study displayed low-levels of resistance to penicillin, in line with other studies (Zhao et al., 2017). Serotypes 15B/15C, 19A, and 15A showed the highest non-susceptibility to penicillin and this finding has been documented in other studies (Lee et al., 2013; Yahiaoui et al., 2018). The prevalence of erythromycin-non-susceptible pneumococci in the current study was 18%, which was higher than that reported in The Gambia (0%) (Hill et al., 2006) and in Fiji (11.6%) (Russell et al., 2006) but lower than 37% reported in India (Coles et al., 2002). Factors associated with high antibiotic-non-susceptibility in the current study could not be determined due to the limited sample size but is addressed in detail in another manuscript under review using a larger sample size (*n* = 800).

Compared to penicillin and erythromycin, the prevalence of cotrimoxazole-non-susceptible isolates in this study was high (45%). Russell et al. (2006) reported a relatively low cotrimoxazole non-susceptibility level of 20.3% among Fijian children within the same age group. However, high cotrimoxazole non-susceptibility rates have been reported among Gambian (60%) (Hill et al., 2006) and Indian (81%) (Coles et al., 2002) children less than one year of life. In South Africa, cotrimoxazole is given as prophylaxis to individuals who are HIV-infected or exposed. Infants are administered cotrimoxazole prophylaxis from 4 or 6 weeks of life until the child is confirmed HIV-uninfected (Moodley et al., 2013). Twenty-four percent (33/137) of the infants in this study were born to mothers who were HIV-infected, which may have contributed to the high levels of cotrimoxazole non-susceptibility observed. The acquisition of cotrimoxazole-on-susceptible pneumococci from the mother or other sources cannot be disregarded since cotrimoxazole is widely used in low and middle income countries including South Africa (Church et al., 2015).

None of the selected pneumococcal isolates were non-susceptible to ciprofloxacin or levofloxacin. Fluoroquinolones, particularly, levofloxacin, gatifloxacin, and moxifloxacin are increasingly used for the treatment of community-acquired pneumonia (Mandell et al., 2007), especially in high income countries where tuberculosis is less prevalent (Grossman et al., 2014). These antibiotics are uncommonly used in the public health sector, including the clinics and hospital that our population accessed. However, non-susceptibility to levofloxacin has emerged in several countries and this poses a threat to its ability to treat resistant pneumococcal infections (Vasoo et al., 2011; Cho et al., 2012; Ba et al., 2014; Themelidis et al., 2014; El-Nawawy et al., 2015).

This study showed that PCV13 serotypes had higher proportions of antibiotic-non-susceptibility than non-PCV13 serotypes, and this has been observed elsewhere (Lee et al., 2010; Kim C.J. et al., 2016). However, in the current study, this might be attributable to the time elapsed between PCV13 implementation in 2011 (in South Africa) and the commencement of the study in 2012. Interestingly, antibiotic-non-susceptibility among non-typeable pneumococci was also frequent. Although non-typeable pneumococci have limited potential for disease, they may act as a reservoir for the transfer of antibiotic-resistance elements to other bacteria. Monitoring of resistance levels and colonization rates of non-typeable pneumococci is therefore important (Marsh et al., 2010).

Few studies have reported on the carriage duration of antibiotic-non-susceptible pneumococci, with most focused on individuals with respiratory tract infection (Ekdahl et al., 1997; Gunnarsson and Ekdahl, 1998; Högberg et al., 2007). The carriage duration of penicillin-non-susceptible pneumococci (mean 18 days) in this study was lower than that reported among Swedish infants with respiratory tract infection (mean 49 days) (Ekdahl et al., 1997). In this study, duration of carriage was related to both serotype (PCV-13 vs. non-PCV-13) and antibiotic non-susceptibility. Firstly, we observed that penicillin or erythromycin-non-susceptible pneumococci were carried for a shorter duration than penicillin or erythromycin-susceptible pneumococci. This may suggest that a fitness cost could be associated with expression of antibiotic-resistance (Lees et al., 2017; Lehtinen et al., 2017). Our finding was, however, dependent on serotype since penicillin, erythromycin or cotrimoxazole-non-susceptible PCV-13 serotypes were carried for a longer duration than susceptible PCV-13 serotypes, while the inverse was true for non-PCV-13 serotypes. Penicillin-non-susceptible serotype 19F and erythromycin-non-susceptible serotypes 19F and 9V were carried for a longer duration than the other PCV13 serotypes. One possible explanation for our findings is that, in pneumococci, the relative fitness cost of particular non-susceptible genotypes may depend on the genetic background of the strains in which the resistance-conferring mutations or genes are found (Lees et al., 2017). Further work, using whole genome sequencing to identify the resistance-conferring genes and strain genetic background is needed.

Dual resistance and MDR were generally low, however, significantly higher rates of dual resistance were observed among PCV13 serotypes compared to non-PCV13 serotypes for penicillin-cotrimoxazole (12% vs. 5%, *p* = 0.003) and erythromycin-cotrimoxazole (11% vs. 4%, *p* = 0.001). Penicillin-non-susceptible pneumococci have been shown to be frequently non-susceptible to other classes of antibiotics (Linãres et al., 1996). In the current study, penicillin-non-susceptible pneumococcal isolates were frequently non-susceptible to erythromycin and cotrimoxazole, as described elsewhere (Critchley et al., 2000; Liñ Ares et al., 2010; Stacevičienė et al., 2016).

This study documented shifts in the antibiotic susceptibility profiles over time within the same pneumococcal serotype carried by an infant. Seventy-six percent (79/104) of the shifts observed were for penicillin susceptibility profiles, however, since genetic resistance determinants were not investigated, we are unable to confirm whether switches in resistance occurred within the same strain, or identify the genetic basis of resistance acquisition or loss, and this should be addressed in future studies.

There were several limitations to this study. Firstly, pneumococcal isolates were only screened against five commonly used antibiotics. Secondly, the infants enrolled were from the same geographic district, therefore the results may not be generalizable. This cohort included only vaccinated infants, and no comparison to unvaccinated children could be made. The methods used in this study were unable to detect co-colonization with multiple serotypes or confirm or characterize genetic resistance determinants. A further limitation is the inability to determine risk factors associated with antibiotic non-susceptibility and carriage duration of non-susceptible pneumococci due to the small sample size. This study does, however, provide baseline data on the prevalence, trends, and carriage duration of antibiotic-non-susceptible pneumococci among infants enrolled in a PCV13 immunized birth cohort in a low and middle income country setting with a high incidence of lower respiratory tract infection in infants (Zar et al., 2016).

## Conclusion

In conclusion, this study showed that the NP carriage of antibiotic-non-susceptible pneumococci was relatively constant through the first year of life. Despite a high vaccine coverage, PCV13 serotypes were identified and were more commonly non-susceptible to penicillin, erythromycin, and cotrimoxazole. Overall, penicillin or erythromycin-non-susceptible pneumococci were carried for a shorter duration than susceptible pneumococci, however, non-susceptible PCV13 serotypes were carried for a longer duration than non-susceptible non-PCV13 serotypes.

## Author Contributions

MN, FD, CM, and HZ conceptualized and supervised this study. MN, FD, and HZ obtained funding. RM and FD performed the experiments and analyzed the data. HZ, CM, SA, FR, and MN contributed to supervision, experimental design, data analysis and manuscript preparation. All authors reviewed, contributed to, and approved the final manuscript.

## Conflict of Interest Statement

The authors declare that the research was conducted in the absence of any commercial or financial relationships that could be construed as a potential conflict of interest.

## Supplementary Material

Figure S1 Point-prevalence of multiple antibiotic-non-susceptible pneumococci (*n* = 1520) obtained from 137 infants throughout the first year of life. MDR, Multidrug-resistance; Pen G-Ery, Penicillin G and erythromycin; Pen G-Sxt, Penicillin G and cotrimoxazole; Ery-Sxt, Erythromycin and cotrimoxazole. The frequency of MDR was high late in the first year of life while penicillin G and cotrimoxazole dual resistance was common early in life.

Figure S2 Carriage duration of the most frequently isolated serotypes among 137 infants according to antibiotic susceptibility, **(A)** penicillin, **(B)** erythromycin, and **(C)** cotrimoxazole. PCV13 serotypes, Serotypes included in the 13-pneumococcal conjugate vaccine; Non-PCV13 serotypes, Serotypes not included in the 13-pneumococcal conjugate vaccine.
