# Supplementary figures and images for: Nasopharyngeal Carriage of Antimicrobial-Resistant Pneumococci in an Intensively Sampled South African Birth Cohort
Source: Front Microbiol. 2019 Mar 27;10:610. doi: 10.3389/fmicb.2019.00610 (PMC6446970; doi:10.3389/fmicb.2019.00610)

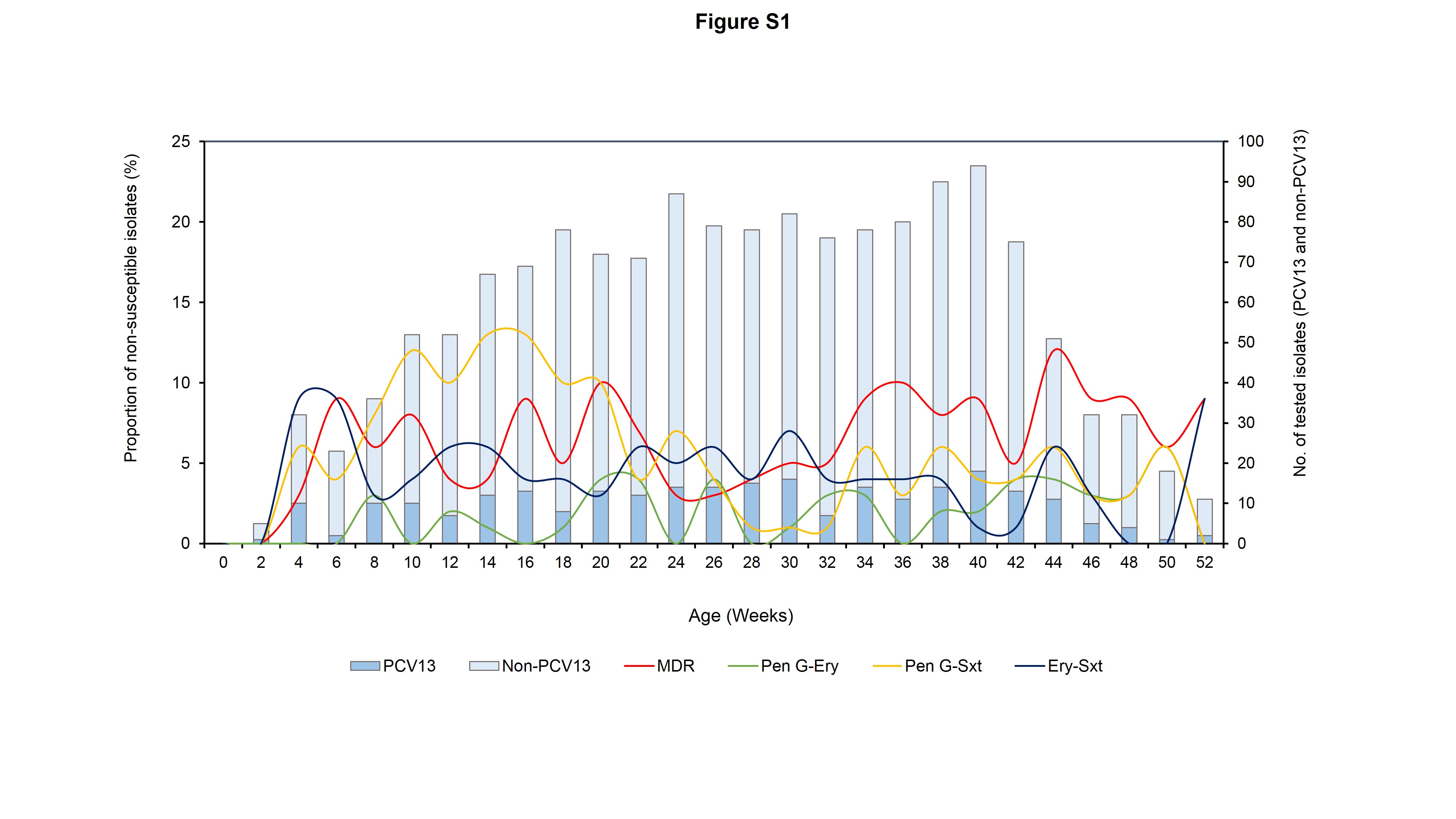

Supplement: Figure S1 — Point-prevalence of multiple antibiotic-non-susceptible pneumococci (n = 1520) obtained from 137 infants throughout the first year of life. MDR, Multidrug-resistance; Pen G-Ery, Penicillin G and erythromycin; Pen G-Sxt, Penicillin G and cotrimoxazole; Ery-Sxt, Erythromycin and cotrimoxazole. The frequency of MDR was high late in the first year of life while penicillin G and cotrimoxazole dual resistance was common early in life. [file Image_1.JPEG]

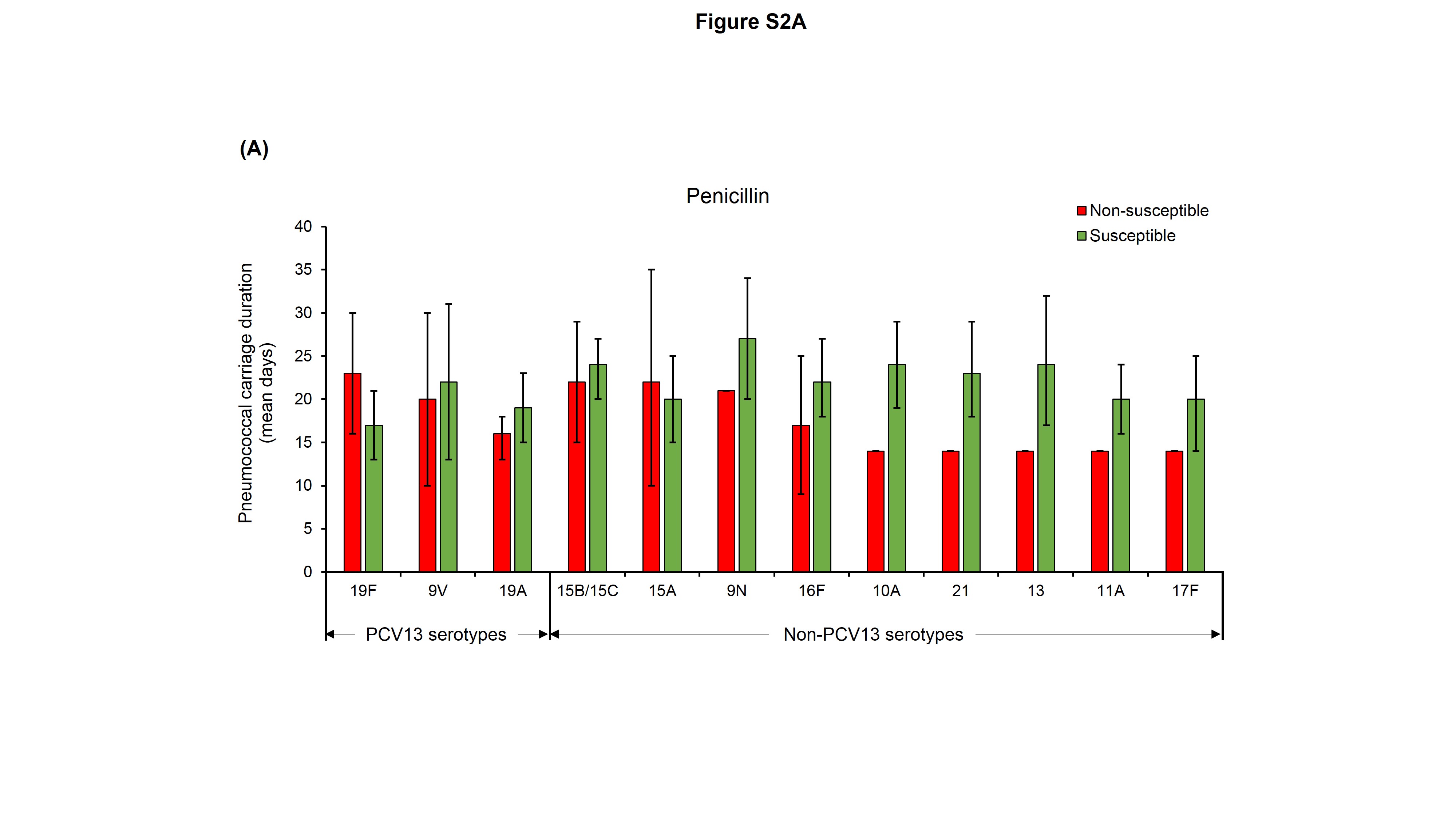

Supplement: Figure S2 — Carriage duration of the most frequently isolated serotypes among 137 infants according to antibiotic susceptibility, (A) penicillin, (B) erythromycin, and (C) cotrimoxazole. PCV13 serotypes, Serotypes included in the 13-pneumococcal conjugate vaccine; Non-PCV13 serotypes, Serotypes not included in the 13-pneumococcal conjugate vaccine. [file Image_2.JPEG]

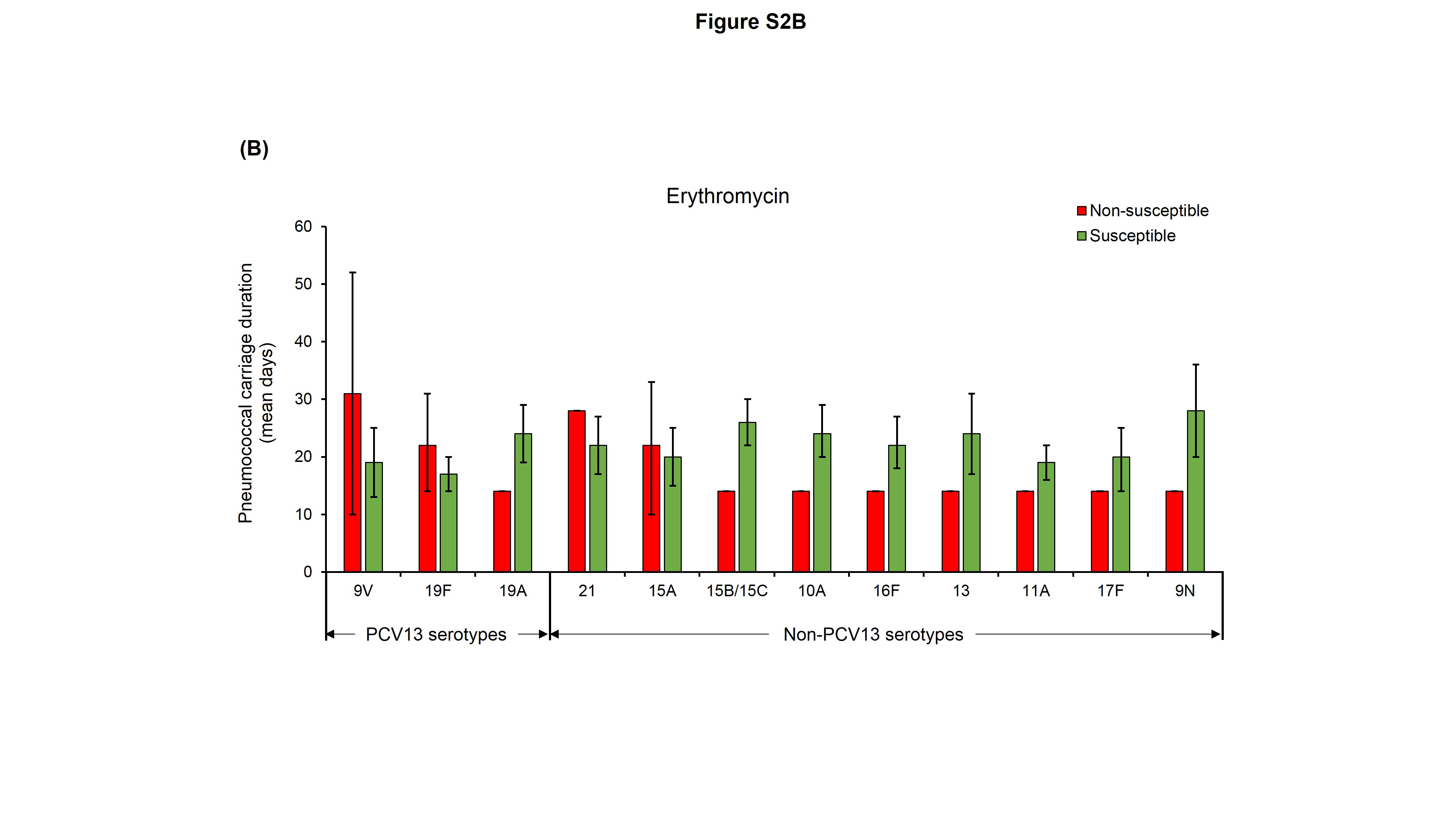

Supplement: Supplementary file 3 [file Image_3.JPEG]

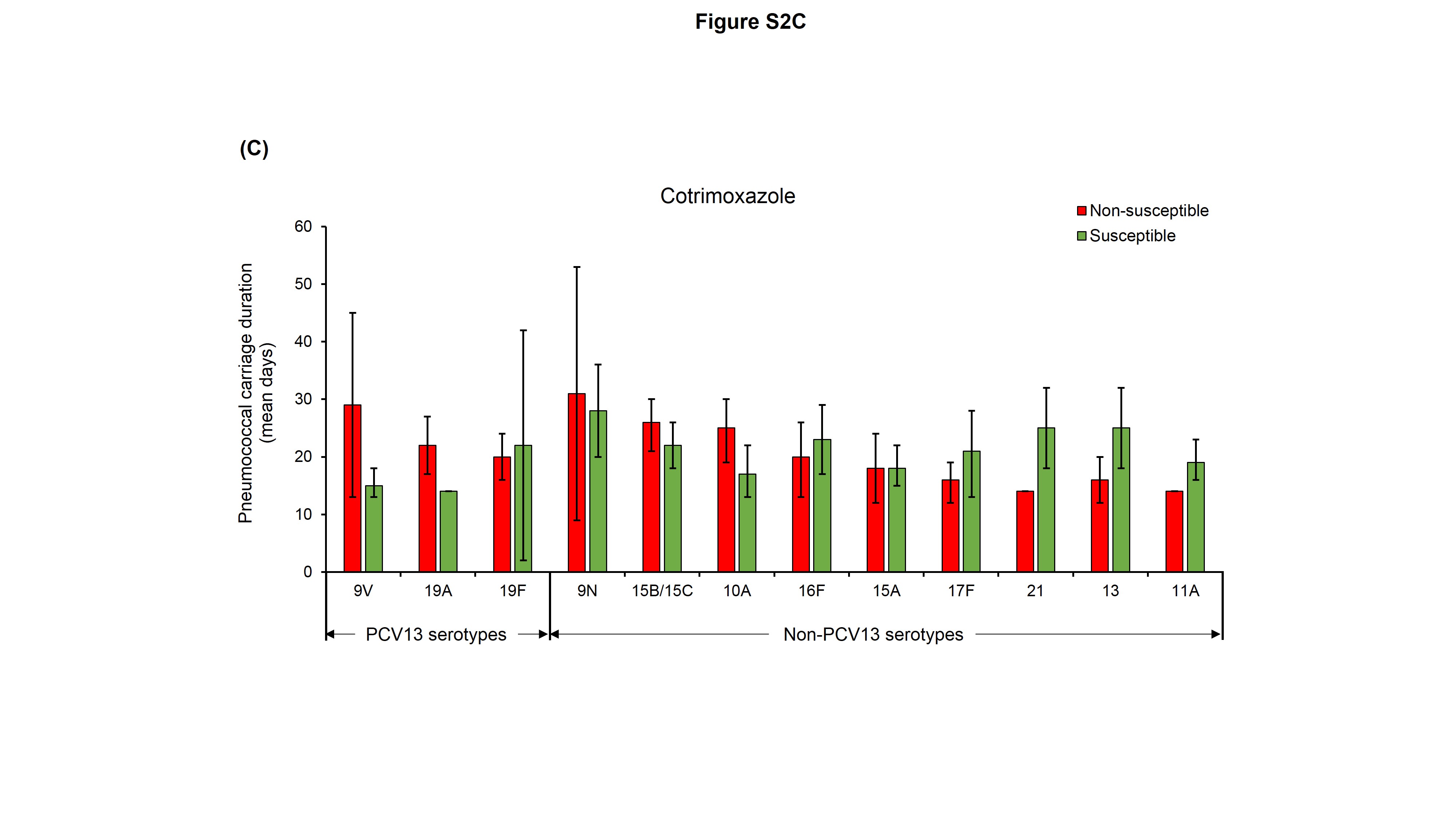

Supplement: Supplementary file 4 [file Image_4.JPEG]
