# Supplementary material for: Nasopharyngeal Carriage of Antimicrobial-Resistant Pneumococci in an Intensively Sampled South African Birth Cohort
Source: Front Microbiol. 2019 Mar 27;10:610. doi: 10.3389/fmicb.2019.00610 (PMC6446970; doi:10.3389/fmicb.2019.00610)
Supplement: Supplementary file 5 [file Table_1.docx]

**Table S1:** Carriage of antibiotic-resistant pneumococcal isolates by serotype

| Serotype | n | Number of non-susceptible isolates | | | | | | | | |
| --- | --- | --- | --- | --- | --- | --- | --- | --- | --- | --- |
|  |  | Non-susceptibility to: | | |  | Dual resistance to: | | |  | MDR |
|  |  | Pen | Ery | Sxt |  | Pen-Ery | Pen-Sxt | Ery-Sxt |  |  |
| PCV13 serotypes | | | | | | | | | | |
| 19A | 41 | 13 | 5 | 31 |  | 1 | 8 | 1 |  | 3 |
| 19F | 26 | 8 | 7 | 19 |  | 0 | 3 | 3 |  | 4 |
| 9V | 20 | 3 | 9 | 11 |  | 0 | 1 | 6 |  | 2 |
| 6A | 19 | 1 | 2 | 8 |  | 0 | 0 | 1 |  | 1 |
| 23F | 8 | 2 | 5 | 6 |  | 0 | 0 | 3 |  | 2 |
| 6B | 6 | 4 | 2 | 3 |  | 1 | 1 | 0 |  | 1 |
| 3 | 6 | 1 | 0 | 2 |  | 0 | 1 | 0 |  | 0 |
| 18C | 3 | 0 | 0 | 0 |  | 0 | 0 | 0 |  | 0 |
| 14 | 2 | 2 | 0 | 2 |  | 0 | 2 | 0 |  | 0 |
| 4 | 2 | 0 | 0 | 0 |  | 0 | 0 | 0 |  | 0 |
| Total (%) | 133 (100) | 34 (26) | 30 (23) | 82 (62) |  | 2 (2) | 16 (12) | 14 (11) |  | 13 (10) |
| Non-PCV13 serotypes | | | | | | | | | | |
| 15B/C | 91 | 20 | 13 | 57 |  | 1 | 9 | 2 |  | 10 |
| 10A | 40 | 7 | 6 | 25 |  | 1 | 3 | 2 |  | 3 |
| 21 | 37 | 8 | 7 | 12 |  | 3 | 2 | 1 |  | 3 |
| 16F | 36 | 5 | 5 | 15 |  | 0 | 1 | 1 |  | 4 |
| 13 | 31 | 5 | 7 | 8 |  | 0 | 1 | 2 |  | 4 |
| 15A | 30 | 10 | 10 | 14 |  | 5 | 1 | 1 |  | 4 |
| 11A | 27 | 1 | 2 | 10 |  | 0 | 0 | 2 |  | 0 |
| 17F | 22 | 1 | 1 | 8 |  | 1 | 0 | 0 |  | 0 |
| 9N | 22 | 3 | 2 | 5 |  | 0 | 1 | 0 |  | 1 |
| 7C | 19 | 3 | 3 | 6 |  | 0 | 0 | 0 |  | 3 |
| 35B | 17 | 3 | 1 | 8 |  | 0 | 1 | 0 |  | 1 |
| 35B/C | 15 | 5 | 1 | 6 |  | 1 | 4 | 0 |  | 0 |
| 15B | 13 | 4 | 3 | 6 |  | 0 | 1 | 0 |  | 2 |
| 17A | 10 | 1 | 1 | 4 |  | 0 | 0 | 0 |  | 1 |
| 23A | 10 | 0 | 0 | 2 |  | 0 | 0 | 0 |  | 0 |
| 31 | 8 | 1 | 1 | 1 |  | 0 | 0 | 0 |  | 1 |
| 35F/47F | 8 | 1 | 1 | 2 |  | 0 | 1 | 0 |  | 0 |
| 34 | 7 | 0 | 1 | 1 |  | 0 | 0 | 1 |  | 0 |
| 35A | 6 | 1 | 2 | 1 |  | 1 | 0 | 1 |  | 0 |
| 35F | 5 | 0 | 0 | 1 |  | 0 | 0 | 0 |  | 0 |
| 24F | 5 | 1 | 3 | 4 |  | 0 | 0 | 2 |  | 1 |
| 22F | 5 | 0 | 0 | 0 |  | 0 | 0 | 0 |  | 0 |
| 8 | 4 | 0 | 0 | 0 |  | 0 | 0 | 0 |  | 0 |
| 12B | 4 | 0 | 0 | 1 |  | 0 | 0 | 0 |  | 0 |
| 18B | 4 | 0 | 1 | 0 |  | 0 | 0 | 0 |  | 0 |
| 22A | 4 | 0 | 1 | 1 |  | 0 | 0 | 1 |  | 0 |
| 23B | 4 | 0 | 1 | 3 |  | 0 | 0 | 1 |  | 0 |
| 33A | 4 | 1 | 1 | 4 |  | 0 | 1 | 1 |  | 0 |
| 33C | 4 | 1 | 1 | 3 |  | 0 | 1 | 1 |  | 0 |
| Others | 36 | 3 | 3 | 10 |  | 1 | 1 | 1 |  | 1 |
| Total (%) | 528 (100) | 85 (16) | 78 (15) | 218 (41) |  | 14 (3) | 28 (5) | 20 (4) |  | 39 (7) |
| NT (%) | 99 (100) | 28 (28) | 28 (28) | 44 (44) |  | 5 (5) | 6 (6) | 2 (2) |  | 16 (16) |
| Overall (%) | 760 (100) | 147 (19) | 136 (18) | 344 (45) |  | 21 (3) | 50 (7) | 36 (5) |  | 68 (9) |

Pen- Penicillin, Ery-Erythromycin, Sxt- Cotrimoxazole, MDR- Multidrug resistance, n- number. PCV13 serotypes- Serotypes included in the 13-pneumococcal conjugate vaccine, Non-PCV13 serotypes- Serotypes not included in the 13-pneumococcal conjugate vaccine, NT- Non-typeable. Others include serotypes 20, 38, 11F, 18A, 19B, 19C, 22A/F, 23B, 25A/38, 33B/35C, 33F, 47F, and 6C/D.
